# Video Remote Sign Language Interpreting in Health Communication for Deaf People: Protocol for a Randomized Controlled Trial

**DOI:** 10.2196/64590

**Published:** 2024-12-02

**Authors:** Minerva Rivas Velarde, Laura Catalina Izquierdo Martinez, Jyoti Dalal, Angela Martinez-R, Danna Lesley Cruz Reyes, Jess Cuculick, Alexie Vallejo-Silva, Jonathan Irreño-Sotomonte, Nora Groce

**Affiliations:** 1 School Of Health Science University of Applied Sciences and Arts Western Geneva Switzerland; 2 Institute of Ethics History and Humanities Faculty of Medicine University of Geneva Geneva Switzerland; 3 Universidad del Rosario School of Medicine and Health Sciences Bogotá Colombia; 4 Faculty of Medicine University of Geneva Geneva Switzerland; 5 National Technical Institute for the Deaf Rochester, NY United States; 6 Clinica Nuestra Señora de la Paz Bogota Colombia; 7 Institute of Epidemiology & Health University Collegue London London United Kingdom

**Keywords:** assistive technology, video remote interpretation, deaf, disability, communication, healthcare

## Abstract

**Background:**

The current standard of interpretation provision is not efficacious or not acceptable to Deaf patients who communicate using sign language. In-person or video relay interpretation (VRI) sign language interpretation is largely unavailable. There are no clear data on the availability of VRI or in-person interpretation. Given the limited number of available sign language interpreters and the cost, VRI may be more available than in-person. Existing evidence tends to focus on assessing personal preferences of Deaf users regarding interpretation and interpreters’ preferences. Although respecting preferences is essential, there is a vacuum of knowledge on how the format of access to interpretation impacts the quality of communication between Deaf persons and health personnel.

**Objective:**

This study aims to look at the effectiveness of the VRI system in improving communication outcomes between Deaf patients and doctors versus the available standard of care of the usual communication tools, including informal interpretation, lip- or note-reading, and using their mobile phones to contact a formal or informal interpreter, for Deaf patients aged 18 years and older in Bogota, Colombia.

**Methods:**

This is a randomized controlled trial with a total sample size of 216 participants, divided into 2 groups: an intervention group, which receives a medical appointment using VRI, and a control group, which receives a medical appointment using standard communication. Both the Deaf participants and the health care professionals will be blinded to the allocation, as they will not know whether the appointment will involve VRI or standard communication until they arrive at the office. The primary outcome measure will be an assessment of communication using a Doctor-Patient Communication Scale. This scale was translated into Colombian Sign Language following a rigorous cultural adaptation and translation procedure. Furthermore, the database contains key clinical variables and recommendations provided by the doctor during a general medicine appointment. We will compute associations.

**Results:**

Recruitment opened on August 24, 2023. As of July 2024, 180 participants had been enrolled. The intervention and data collection were finalized in October 2024. The findings of this study are expected to be submitted for publication in early 2025.

**Conclusions:**

This study will provide rigorous evidence regarding information and communications technology intervention in health care, addressing empirical challenges in using inclusive research designs in public health. In addition, effective VRI models that address the challenges faced by Deaf people will be tested, implemented, and maintained in low- and middle-income countries. A disability-inclusive evaluative tool for quality communication mediated by VRI in health care is also tested. Ultimately, this will lead to evidence-based recommendations for implementing the Convention on the Rights of Persons with Disabilities (CRPD) in mobile health contexts.

**Trial Registration:**

ClinicalTrials.gov NCT05966623; https://clinicaltrials.gov/study/NCT05966623

**International Registered Report Identifier (IRRID):**

DERR1-10.2196/64590

## Introduction

Sign language interpretation is essential for facilitating communication between Deaf people and hearing individuals; it creates a cultural and linguistic bridge. There is growing evidence showing that communication barriers between health care staff members and Deaf patients are the main factors for health disparities. Deaf people continue to experience a lack of access to health care services in their preferred language; there is insufficient health information in sign language and insufficient knowledge and awareness by professionals of their linguistic and cultural requirements [1,2].

The current standard of interpretation provision is not efficacious or not acceptable to Deaf patients who communicate using sign language. In-person or video relay interpretation (VRI) is largely unavailable. There are no clear data on the availability of VRI or in-person interpretation. Given the limited number of available sign language interpreters and the cost, VRI may be more available than in-person interpretation. Existing evidence tends to focus on assessing personal preferences of Deaf users in regard to interpretation and interpreters’ preferences. Thus, there is a vacuum of knowledge regarding whether this format of interpretation succeeds at enabling quality communication, which is core to the purpose of interpretation services. A handful of studies, all from high-income countries (HICs), predominantly the United States, provide some empirical data comparing VRI in-person interpretation [3] and show no statistical difference in their preference for VRI versus in-person interpretation for critical care, but a statistical difference for noncritical care. Myers et al [4] reported that their study participants preferred in-person interpreting due to recurrent technical difficulties with VRI. Kushalnagar et al [5] found that most people in the United States do not have access to VRI, but those with access stated that such access would meet their interpretation needs as much as in-person does [5]. Hall and Ballard [6], using a qualitative exploration of preferences, claim that Deaf people are inclined to prefer in-person interpretation, while noting that such provision is highly constrained due to shortages in the workforce. Assessing the efficiency of VRI versus the standard of care would be of more value, given that there is no other effective intervention to compare.

### Access to Health Care for Deaf People

Access to health care services for Deaf people has historically been poor compared with the hearing population. Deaf people have worse mental and physical health than the hearing population [1,7]. Deaf people are at high risk of developing cardiovascular diseases, diabetes, depression, overweight, and obesity; they also have a higher risk of partner violence and depression, and there is a higher suicide rate [8,9]. They are more prompt to underdiagnosis, and undertreatment of chronic diseases can put them at risk of preventable diseases and potentially reduce their life expectancy [9]. Furthermore, those in low- and middle-income countries (LMICs) are exposed to higher health disparities than those living in HICs [10,11].

### Challenges to the Provision of Sign Language Interpretation

Deaf people are more likely to avoid health care providers, partially because of the lack of means of communication with these providers and available interpreters [12,13]. Thus, even if interpreters are available, the pool of sign language interpreters tends to be relatively small, even in HICs [14]. Shortages are more acute in LMICs, where official certification is often unavailable or limited. For example, in 2015, there were approximately 42 certified sign language interpreters in Mexico [15] and 37 certified interpreters in Paraguay [16]. Sign language interpretation is often a service delivered by people who have completed a certain number of hours in sign language courses and who subsequently present themselves in institutions offering their interpreting services without an official degree and without specific studies that train them in the educational field [17]. The vacuum of available workforce and training is an issue in HICs, too. Deaf people associations in Spain and Canada have called for attention to this issue [18,19]. Furthermore, aside from the availability, the competencies of those interpreting in the health care context are a concern, as specific health communication training is mainly absent [11,20,21]. Significant progress has been made by other countries, like the United States and England, which have stabilized frameworks and regulations and standardized training systems quality standards [22].

Aside from issues of availability and competencies of the sign language interpretation workforce, a second barrier to the interpretation process is the lack of skills in health personnel. Health personnel at large tend to be unaware and unskilled in working with sign language interpreters, which, alongside limited awareness about deafness overall, often results in poor communication and, ultimately, patients not getting the information they need to decide on their health or treatments [14]. Good communication between health personnel is critical to patient experience and quality of care [23]. Furthermore, good communication has positively improved patient health outcomes [24].

One solution to these limitations is the growing availability of VRI, a specialized translation service that relies on a high-speed internet connection and a camera to connect a remote sign language interpreter with a health care professional and a Deaf patient to facilitate their communication [5]. VRI technology is not subject to geographical or time limitations, as the interpreter can provide their services from anywhere without having to travel to the appointment. As sign language interpreters are mainly unavailable due to workforce constraints, building capacities in this sector will take many years or generations, especially in LMICs. Therefore, VRI can be a good solution for enabling communication between health care staff members and Deaf people.

### Trial Objectives and Purpose

Available data tend to focus on the evaluation of the personal preferences of Deaf users regarding interpretation, as well as the preferences of interpreters. This is useful, but knowing whether these preferences translate into better communication and health services relationships and outcomes is unknown. Furthermore, assessing the feasibility of in-person services, scalability, and sustainability are often left behind. Hall and Ballard [6] show that Deaf people prefer on-site interpretation, acknowledging the workforce shortage and operational challenges in the short term in the United States [6]. From a different angle, in Colombia, Izquierdo et al (unpublished data, 2024) documented that in tight-knit Deaf communities, some people prefer VRI, reporting a greater feeling of privacy in health-related settings. As evidence grows, and the interpretation workforce may also increase and mature in their competencies and regulation, it is pertinent to ask if VRI technology could enable health systems to make the most of the available workforce and enable Deaf communities to communicate effectively with health personnel. There are no baseline data on this issue nor evidence of the effectiveness of the technology compared with the standard of care, which includes informal interpretation, lip-reading or note-taking, or using their mobile phones to contact a formal or informal interpreter. A total of 80% of Deaf people live in LMICs; therefore, running a study in such a setting shall have more global implications.

This study is being conducted in Colombia. The following criteria were used to select this country: (1) it is a signatory country to the Convention on the Rights of Persons with Disabilities (CPRD); (2) it has demonstrated legislation, commitment, and progress of being a champion for inclusive information and communications technology (ICT; G3ICT and Zero Project International Telecommunication Union); (3) it has the necessary infrastructure to test a working model for VRI in the health care context, which includes good internet coverage throughout the country, availability of VRI for general use, and a workforce of local sign language interpreters with expertise in VRI; (4) is a high- to middle-income country, and variations within its territory are relevant for both LMICs and HICs; and (5) Deaf peoples’ organizations have interest in this study and have infrastructure that could support both the survey and the intervention. The researchers are working in partnership with local and international disabled people organizations. The Ministry of Health and Universidad del Rosario are the implementing partners.

## Methods

### Design Based Upon the Real-Life Context in Colombia

This study trial is part of a larger study that focuses on understanding structural and societal factors alongside impairments to determine how an individual experiences health and disability (Swiss National Science Foundation, PZ00P1_186035). A steering committee governs the design of the study. The committee includes local and international Deaf organizations, leading disability and global health scholars, and public administration representatives.

A nuanced understanding of disability, which considers lived experiences, cultural differences, and intersectionality issues, is essential to inform research on the effectiveness of public health interventions. For instance, examining whether Deaf people and health providers could enable access to VRI and, therefore, mobile devices with video capabilities and the internet requires analysis at multiple levels. In the first part of the study, we ran a health priority assessment through a national e-survey in Colombian Sign Language. This survey rigorously linguistically and culturally adapted research instrument design to measure key indicators and health priorities of the Deaf community and contribute to understanding health inequities by using population comparison. Then, using qualitative interviews, the team looked at the individual-level determinant factors such as technology literacy, device adequacy, affordability, and availability. Then, we collected data on infrastructure, workforce, and health policies in tandem with interviews with health workers and sign language interpreters. This first part revealed where and how exclusion occurs within and outside the health system. Understanding their specific health care needs is paramount. This exploration enables us to determine the most effective model for such interventions, and taking action to improve VRI use across health care systems is a critical step. The design intervention addressed was developed to provide sign language with a fully qualified sign language interpreter, although formal training on health is not available in the country and region. Significant expertise in the health sector was a prerequisite to act as an interpreter in the study. Accessibility issues from the first point of contact with the health setting were considered. The design of the VRI randomized control trial (RCT) study responded to real-life contexts in Colombia.

### Participants in the Sample

This study is an RCT with a total sample size of 216 participants, divided into 2 groups: an intervention group, which receives a medical appointment using VRI, and a control group, which receives a medical appointment using the standard communication (informal interpretation, lip-reading, note-taking, or the use of mobile phones). Both the Deaf participants and the health care professionals will be blinded to the allocation, as they will not know whether the appointment will involve VRI or standard communication until they arrive at the office.

### Intervention Group

The participants were able to learn about the study through social media, advocacy organizations, and word-of-mouth. A researcher, along with a sign language interpreter, contacts each potential participant through a WhatsApp video call (the key communication channel among Deaf organizations in Colombia, using sign language) to explain the project and schedule a medical appointment. Upon arrival at the hospital, participants are greeted by a research assistant who facilitates their admission process. The participants were then guided to the consultation room.

The participants engage in a standard medical consultation known in the Colombian health system as “standard promotion and disease prevention or annual check” using VRI as the means of communication. During the consultations, the health care professional conducts a medical history assessment, performs a physical examination, and provides education and recommendations based on the findings.

The VRI is used during the appointment. A 32-inch screen displays the Colombian Sign Language interpreter, who connects through the Zoom (Zoom Video Communications) platform. A professionally accredited Colombian Sign Language interpreter with robust experience in medical interpretation facilitates communication between the health care professional and the Deaf participant during the consultation.

After the medical consultation, participants complete the Doctor-Patient Communication (DPC) scale. This scale is administered by a research assistant using a tablet and is available on the LimeSurvey platform with Spanish subtitles and Colombian Sign Language videos.

### Control Group

The participants in the control group gain access to the same intervention, albeit without VRI contact. They communicate with the doctor using standard communication methods (eg, self-arranging interpretation, lip-reading, note-taking, or the use of images).

### Explanation for Choice of Comparator

In-person or VRI sign language interpretation is largely unavailable. Thus, there are no clear data on the magnitude of the availability gap of VRI or in-person interpretation. In-person qualified sign language interpretation in the health care setting is considered the ideal service provision standard. Thus, it is mainly unavailable even in HICs. Furthermore, the assumption is based upon minimal available evidence on the personal preferences of Deaf people in the United States. There is no evidence that in-person interpretation is efficient in the context of weaker regulation and infrastructure, such as low sign language proficiency rates among Deaf people, lack of standard qualification of interpreters, and lack of interpreters and sustainable financing. To our knowledge, no study assesses DPC while using sign language interpretation.

Given the cost, VRI may be more sustainable than in-person care. Given that there is no other effective intervention to compare, assessing the efficiency of VRI versus the standard of care would be of greater value.

### Sample Size

This study includes 216 participants who were randomized into 2 groups. As no clinical trials in the field have been undertaken, there are no previous effect sizes to consider. However, 216 participants are deemed sufficient to find clinically significant differences.

To establish that the control group that uses VRI improves concerning the variables of interest, we followed the superiority approach and computed the sample size needed using the equation given below:



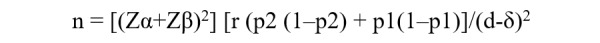



Where Zα=1.64 is the *z* score considering the level of significance to be 5%, α =.05; Zβ=0.84 is the *z* score for 80% power, β = 0.2; δ=0.05 is the superiority margin (5%); p1=0.85 is the proportion in the intervention group; p2=0.65 is the proportion in the control group; d=p1–p2 is the difference between the true groups, which is 0.20; r=1 is the ratio of the sizes of the 2 groups (considering the same size for both the groups); and n is approximately 97.

Consequently, under these conditions, we expect 10% of the enrolled participants to drop out due to any circumstances that may arise and therefore not be analyzable. The final sample size was 100×(2n)/(100–10), about 216.

### Inclusion, Exclusion, and Withdrawal Criteria

#### Inclusion criteria

To be included in the study, the participants must reside in Bogotá, Colombia, be aged at least 18 years, be fluent in Colombian Sign Language, and have the availability to attend the medical appointment. In addition, they must possess sufficient sensorimotor, cognitive, and communication skills to interact with health care personnel independently.

#### Exclusion criteria

Participants are excluded if they have additional impairments that affect language development or the use of sign language.

#### Withdrawal

Participation can be withdrawn during the treatment period without specifying a reason behind the decision.

### Safety Monitoring and Reporting

The study has been registered in ClinicalTrials.gov under the identifier NCT05966623 since November 29, 2023. The data being stored will be encrypted, and all participants will receive an identification code, thus ensuring the anonymity of the participants during the analysis of the results. The LimeSurvey website is publicly accessible and allows for a wider distribution of surveys. The University of Geneva and Universidad del Rosario, partners in this research, are institutionally subscribed to this platform. The service provided by the platform meets the data protection and security requirements mandated by the relevant legal frameworks and ethical standards in Colombia and Switzerland. Furthermore, the transfer and storage of data comply with security guidelines. Access to the collected data is strictly restricted to research team members.

### Assignment of Interventions: Blinding

To prevent selection bias, the participants will be assigned to the intervention or control group using random assignment techniques with block randomization. The randomization of participants will allow for the evaluation of treatment effects while ensuring that the observed outcomes are truly due to the treatment and not influenced by other factors. Using blocks in random assignment is a common strategy to ensure that the groups are balanced in terms of relevant characteristics, which helps minimize variability and potential biases in assigning patients to the study groups. In this case, a randomization list will be created using the website randomization.com. This site will facilitate random assignments using the block method to ensure a balanced allocation of patients between the 2 groups.

A defined procedure will be followed to define the block randomization lists.

First, determining the block size: a block size of 4 will be defined (2 patients for each group). This block size is chosen because it is a multiple of the number of groups, which in this case are 2 (intervention and control). Having a block size that is a multiple of the number of groups ensures that each group is equally represented within the blocks, which aids in maintaining a balanced random assignment of patients to the intervention and control groups. This helps ensure the validity and reliability of the study results.

Second, randomly assigning each participant within a block to a group: for example, with a block size of 4 and 2 groups (control and intervention, A and B), the assignment could be as follows:

- Block 1: ABBA
- Block 2: BAAB
- Block 3: ABAB
- Block 4: BABA

This process continues until all participants have been assigned to the different groups. To maintain concealment, the block sizes will not be revealed.

Once the participants enter the medical appointment, they will receive a box containing several envelopes. Each participant will select an envelope that will provide their participant code and the corresponding group designation (control or intervention). The researcher, the participant, the general practitioner, and the sign language interpreter will only learn the group to which the participant belongs when the envelope is opened upon entering the medical consultation.

### Primary Outcomes

The primary outcome measure will be an assessment of communication using the DPC. The DPC [25] is made up of 13 questions. Each question has 4 response options, which are no, possibly no, possibly yes, and yes. The scale has high internal consistency (Cronbach α=.89) and good external validity. This is an easy-to-use and validated generic questionnaire to assess communication in the context of acute conditions, usable both in clinical research and in routine practice, which measures (1) creating a good interpersonal relationship, (2) exchanging information, and (3) making treatment-related decisions that involve the patients in decision-making. This scale was translated into Colombian Sign Language following a rigorous cultural adaptation and translation procedure [26]. The videos are hosted on the LimeSurvey platform in Colombian Sign Language, written in Spanish, and played on a tablet. The participants complete the DPC scale once they leave the medical consultation.

### Secondary Outcomes

The database contains clinical variables such as age, sex, place of birth, BMI, blood pressure, heart rate, respiratory rate, personal medical history (hypertension, diabetes, alcohol consumption, tobacco, and physical activity), use of hearing aids, questions on mental health, presence of hypertension, obesity, and cardiopulmonary, musculoskeletal, and metabolic disorders, recommendations provided by the doctor during a general medicine appointment, and so on. We will compute associations.

### Analyses

The participant demographics will be described both by group and overall sample. Responses for the scale were combined into 2 nominal categories (“yes, probably yes/no, probably no).

We will compute associations among the variables, such as the presence of different disorders, mental health of patients, current lifestyle, and past medical history, for both the groups, using both classic statistical methods (using the Fisher exact test) and machine learning (ML) algorithms, such as association rule learning, and then we will draw a comparison between these two approaches. We will also build suitable ML models (for instance, a regression model depicted below) to predict the occurrence of hypertension, obesity, and cardiopulmonary, musculoskeletal, and metabolic disorders based on the patient’s medical history (hypertension, diabetes, alcohol consumption, tobacco, and physical activity), place of birth, BMI, blood pressure, heart rate, respiratory rate, use of hearing aids, mental health and so on, for both the groups.

Response =predictor+age+gender+place of birth

where “predictor” denotes the patient’s medical history (hypertension, diabetes, alcohol consumption, tobacco, and physical activity), body mass index, blood pressure, heart rate, respiratory rate, use of hearing aids, mental health, and so on; and “response” denotes the occurrence of hypertension, obesity, and cardiopulmonary, musculoskeletal, and metabolic-disorders and so on.

We will report the odds ratios obtained from the regression model, as they are widely used to compare two groups. We will report the predictor-wise odds ratios obtained using the equation given below:

ORs = exp(coefficient of predictor)

### Ethical Considerations

This research has received approval from the Ethics Committee of the University of Geneva in Switzerland (CUREG_ 2021-05-50), the Universidad del Rosario (DVO005 1979-CV1548), and Clínica de Nuestra Señora de la Paz in Colombia (CEI-41-03-2023).

Participation will require written informed consent. During recruitment, the participants will be presented with a detailed information sheet in written Spanish and Colombian Sign Language videos. Each participant will be fully informed about the rationale, objectives, procedures, risks, benefits, and other relevant aspects of the research before enrollment. All documents used will be culturally adapted and translated into Colombian Sign Language.

The project covers the transportation costs for each participant. In addition, an incentive will be provided in the form of a COP 20,000 (US $4.50) voucher, redeemable for groceries at supermarkets.

## Results

This study aims to assess the effects of VRI versus the standard of care (informal interpretation, lip-reading, written notes, and use of images) on communication between health professionals and Deaf people. Recruitment opened on August 24, 2023. The intervention and data collection are expected to be finalized by late October 2024. As of July 2024, 180 participants had been enrolled. The findings of this study are expected to be submitted for publication in early 2025.

## Discussion

### Principal Outcomes

VRI may enable Deaf users to overcome interpretation barriers and potentially improve communication outcomes between them and health personnel within health care services. This protocol envisions to demonstrate that a VRI intervention that is context-sensitive and addresses cultural and infrastructural challenges of health settings in LMICs enables quality communication between Deaf people and nonsigning health personnel. Evidence produced through this RCT will directly address the current knowledge gap regarding the impact of ICTs for communication, particularly sign language interpretation for health care in a case coming from the global south. There is a dearth of evidence in this area.

Available research tends to focus on personal preferences, and quality communication is often not the focus [6], which is problematic. All published literature came from HICs, but most Deaf people live in LMICs [27]. This also means that this literature overlooks the infrastructural constraints and different realities, and such findings have little relevance to LMICs.

To our knowledge, this study is the first clinical trial to examine the effects of VRI and the conventional standard of care, and we anticipate that it will provide significant knowledge about the communication of Deaf people. The study also investigates whether VRI intervention can be beneficial in removing communication barriers in the health care context.

### Strengths and Limitations

The proposed study has several notable methodological strengths. First, it centered on using sign language to identify the Deaf community, drawing apart from traditional biological categorizations that tend to be highly inaccurate, for example, considering people to be Deaf when they have moderate-to-vigorous hearing loss with the use of hearing aids and communicate solely with oral language. This study addresses a linguistic cultural community that encompasses great diversity in terms of hearing functioning, while identifying as Deaf and using Colombian Sign Language as their primary means of communication. It is based upon a participatory research design that includes Deaf representatives from international and national associations, researchers, and policy makers. The study’s design focused on real-life contexts in Colombia and its results will be of more direct relevance to other LMICs, where 80% of Deaf persons live.

This study has some limitations that need to be recognized. First, communication may be more complex in some circumstances, such as critical care and complex medical consultations, such as genetics consultations. Furthermore, persons who are Deaf are at higher risk of ill health and secondary impairments that may impact the capability to interact independently with health care workers or to interact with electronic devices, such as screens for VRI, and this also limits the reach of the study.

### Conclusions

The Deaf community faces multiple communication barriers when accessing the health system. VRI could be a good intervention to implement in the health care context and is being evaluated in a clinical trial. This technology is rolling out worldwide and could bridge the gaps in access to health care for Deaf people. Our findings could contribute to the accessibility and quality of health care for all.

## Abbreviations

CPRD: Convention on the Rights of Persons with Disabilities
DPC: Doctor-Patient Communication
HIC: high-income country
ICT: information and communications technology
LMIC: low- and middle-income country
VRI: video relay interpretation

## Data Availability

The data from this research are stored in the institutional repository of Universidad del Rosario according to the data management plan [28]. The data will be stored with restricted access, and upon the project’s completion, the potential for providing access to relevant data for other researchers will be assessed.
